# Pediatric Oncology Palliative Care Programs in Central America: Pathways to Success

**DOI:** 10.3390/children8111031

**Published:** 2021-11-10

**Authors:** Wendy Cristhyna Gómez García, Silvia Rivas, Gabriela Paz, Marisol Bustamante, Gerardo Castro, Hazel Gutiérrez, Maria Sabina Ah Chu, Pascale Yola Gassant, Rolando Larin Lovo, Yessika Gamboa, Marleni Torres Núñez, Ximena García Quintero, Regina Okhuysen-Cawley

**Affiliations:** 1 Oncology Unit, Hospital Infantil Dr. Robert Reid Cabral, Santo Domingo 10101, Dominican Republic; 2 Palliative Care Program, Unidad Nacional de Oncología Pediátrica, Guatemala 01011, Guatemala; silvia.rivas@unop.org.gt (S.R.); dragabrielapaz@gmail.com (G.P.); mbustamantederuiz@gmail.com (M.B.); 3 Palliative Care, Hospital Escuela, Tegucigalpa 11101, Honduras; gerardoicastrom@yahoo.com; 4 Palliative Care, Hospital Nacional de Niños Dr. Carlos Sáenz Herrera, San José 10103, Costa Rica; hazelmgutierrez@gmail.com; 5 Oncology Unit, Hospital del Niño, Panama City 0816-00383, Panama; mahchu@minsa.gob.pa; 6 Oncology Unit, Hospital St. Damien, Puerto Príncipe HT 6124, Haiti; pascaleyola@hotmail.com; 7 Palliative Care Program, Hospital Nacional de Niños Benjamín Bloom, San Salvador 1101, El Salvador; larinlovo@gmail.com; 8 Oncology Unit, Hospital Nacional de Niños Dr. Carlos Sáenz Herrera, San José 10103, Costa Rica; aygamboacjs@gmail.com; 9 Medical Education, Nicklaus Children’s Hospital, Miami, FL 33155, USA; Marleni.torres@nicklaushealth.org; 10 Palliative Care Program, Fundación Valle de Lili, Cali 760026, Colombia; ximegq@hotmail.com; 11 Divisions of Critical Care Medicine and Palliative Care, Texas Children’s Hospital, Houston, TX 77030, USA

**Keywords:** palliative care, pediatric oncology, pain

## Abstract

Palliative care offers children who have life-limiting and life-threatening oncologic illnesses and their families improved quality of life. In some instances, impeccable symptom control can lead to improved survival. Cultural and financial barriers to palliative care in oncology patients occur in all countries, and those located in Central America are no exception. In this article, we summarize how the programs participating in the Asociación de Hemato-Oncólogos Pediatras de Centro America (AHOPCA) have developed dedicated oncology palliative care programs. The experience in Guatemala, El Salvador, Costa Rica, Panama, Dominican Republic and Haiti is detailed, with a focus on history, the barriers that have impeded progress, and achievements. Future directions, which, of course, may be impacted by the COVID-19 pandemic, are described as well.

## 1. Introduction

The primary goal of palliative care (PC) is to provide comprehensive, holistic (physical, psychological, emotional, social and spiritual) care to children and their family, with the aim of improving quality of life of adults and children with life-threatening and life-limiting illnesses [1], through the prevention and early detection of suffering that is related to the disease and treatment. It follows that many conditions encountered during childhood that lead to health-related suffering could benefit from this approach [2,3].

It has been estimated that 21 million children have conditions which would benefit from palliative care. It is thought that the vast majority of children (>97%) in need of palliative care live in low- and middle-income countries (LMICs) [4], where the availability of Pediatric Palliative Care (PPC) services is limited. There are many barriers to the implementation of PPC programs, including the lack of public health policies, lack of education and training for health professionals, and the availability of essential pain medications in some regions. These are just a few of the challenges that may impede program development. Although the focus of this article focuses on the design and implementation of palliative care for children with cancer, it is well known that a holistic approach to care is of significant benefit to children with other life threatening and limiting disorders, such as complications of prematurity, congenital anomalies, neurologic, and respiratory diseases, such as cystic fibrosis [5].

Palliative care program development has been very uneven internationally; this holds true in the Americas. A systematic review performed in 2017 demonstrated that 66.7% of the countries in South America did not have any PPC activity until 2011, when the first integrated PPC service was documented in that region [6].

Fortunately, though, there has been significant growth in the development of programs in PPC in recent years. For example, the 2019 PC Atlas of Latin America listed 123 teams, corresponding to 0.8 teams/million inhabitants under 15 years of age. Availability of palliative care resources is highly variable: highly developed countries like Uruguay listed 19.3 teams/million inhabitants under 15 years of age, while Paraguay and Venezuela had none.

Eleven PPC teams currently operate in Central America. This includes 3 in Costa Rica, 3 in Panama, 1 in El Salvador, 1 in Guatemala, 1 in Honduras, 1 in the Dominican Republic, and 1 in Haiti [7]. PPCs, including those focused on cancer care, have continued to develop in the region, in parallel with AHOPCA, the Central American association of pediatric hematologists and oncologists. The origins of AHOPCA go back to 1986, when partnerships (“sister” institution relationships) were established between the Manuel de Jesus Rivera “La Mascota” Hospital in Nicaragua and three different institutions in Europe. These strategic partnerships led to the establishment of the Monza International School of Pediatric Hematology-Oncology (MISPHO) a decade later. The goal of MISPHO was capacity-building, through the pillars of training and knowledge transfer, while fostering relationships among health professionals, and between health professionals and community-based sources of support. 

**Figure 1:**
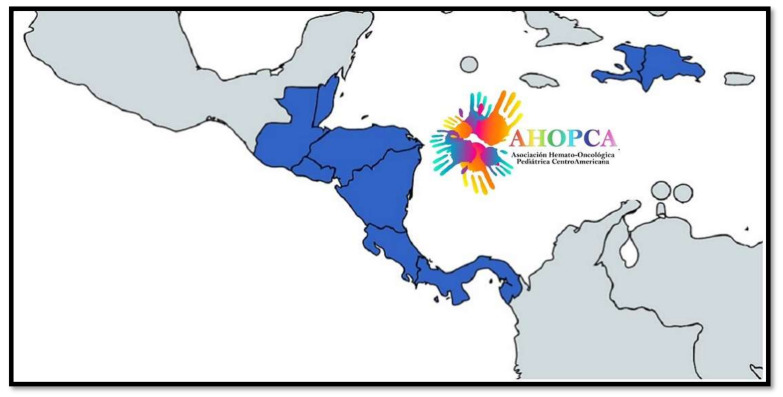
Countries included in AHOPCA.

Many of the palliative care clinicians from Central America met each other for the first time at MISPHO; the Asociación de Hemato-Oncología Pediátrica de Centro América (AHOPCA) was formed in 1998. Guatemala, Honduras, El Salvador, Nicaragua, and Costa Rica were the inaugural members; Panama joined in 2000, the Dominican Republic in 2004 and, finally, Haiti in 2011 (see Figure 1). AHOPCA promoted the development of shared clinical protocols, mainly focused on cancer chemotherapy; educational programs, primarily for physicians and nurses; emphasized the integral role that psychologists and social workers play; and the importance of collaborative research [8].

In the following paragraphs, we describe how palliative care programs were established in each of the countries participating in AHOPCA, and how they impact healthcare delivery at a national level.

## 2. Guatemala

The National Pediatric Oncology Unit (UNOP), inaugurated in 2000 as an individual entity, cares for children with cancer from all over the country. It is subsidized by the Government of Guatemala, with contributions from the private sector.

By 2005, it was evident that oncologists did not have time to properly support patients with poor prognoses. The Department of Onco-pediatric Palliative Care, called “Medicina Integral” (which translates to “Integrative Medicine”) was created to respond to the need to relieve suffering at all levels. This interdisciplinary department began operation with specialists in psychology, social work, child life, palliative care and spiritual care. One of the most important achievements has been a dramatic drop in treatment abandonment, from an all-time high of 40% to only 2%. Medicina Integral, from the outset, has participated in all important medical decision-making discussions for patients with poor prognoses, defined as a less than 50% chance of long-term survival.

This team is also involved in medical decision-making throughout the continuum of care. The need to discontinue chemotherapy in patients displaying a poor overall response to chemotherapy, or to redirect care to comfort-oriented goals, using protocols designed to improve quality of life. In 2016, the Hogar Estuardo Mini hospice was purpose-built to care for these children. A palliative care program outreach program was also created for patients and families who prefer to receive end-of-life care at home. In addition to providing medications for parents to administer at home, 24/7 telephone support was made available, particularly when violence in rural areas precluded home visits.

In 2020, the team participated in 196 curative intent conversations held at the time of diagnosis, 181 conferences were held when patients transitioned from curative intent to palliative, symptom control oriented goals of care, and 200 conferences were held to support patients, families and teams at end-of-life. Overall, 30% of the communications with families were for bereavement purposes. A total of 746 conference calls were conducted to achieve the best possible individualized care.

This model has been operational for approximately 16 years. We continue to approach each day with a spirit of inquiry as we seek to meet our suffering patients’ needs. Our team agrees that although there is still a lot to learn, and room for improvement, we have made some headway. 

The Ammar Ayudando Foundation started out in a parallel fashion in the year 2000, aiming to provide PC to patients with cancer and non-cancer diagnoses receiving care in public hospitals throughout the country. This program focused initially on home care and care provided in public hospitals; shortly thereafter, the first hospice, Villa de la Esperanza was opened to serve Guatemala and neighboring countries in Central America; this hospice is completely free of charge to the poorest patients of the country, and provides care for children and young people with incurable oncological diseases. There has been very close collaboration between the Unidad Nacional de Oncologia Pediatrica (UNOP) since 2000; adult-based programs started in 2015.

Villa de la Esperanza hospice offers services in an environment that strives for compassion and professional excellence, emphasizing the right of human beings to live until the last moment with dignity and love, in harmony with their faith, beliefs, and values, boasting a track record of 21 uninterrupted years of comprehensive palliative care services in Guatemala [9,10].

## 3. El Salvador

The pediatric palliative care program was founded at the Benjamin Bloom National Children’s Hospital (HNNBB) in El Salvador at the beginning of 2008.

The first step in the program was to send a letter to the Ministry of Health asking for the incorporation of several opioids in child-friendly formulations to the list, aiming for improved management of pain and other distressing symptoms. At that time, the Ministry of Health’s list of essential medicines only included morphine for injection, meperidine, nalbuphine, tramadol, tramadol capsules, and acetaminophen/codeine fixed-dose tablets. Medications felt to be essential, and requested by the fledgling palliative care teams, included controlled-release morphine (30 and 60 mg tablets), acetaminophen plus oxycodone (325/5 mg tablet), oxycodone (5 mg tablet), fentanyl (25 micrograms/hour transdermal patches), methadone (10 mg tablet), and tramadol drops (the only oral liquid opioid in the country at that time), since morphine oral solution (and immediate-release tablets) were unavailable. The Ministry of Health officially authorized the inclusion of these drugs in the national essential medicine formulary only 18 months later. 

In 2009, the Palliative Care Unit (PCU) began as an anesthesiology-based service, providing pain and palliative care when a consultation was requested by the oncology department or by the other hospital services for hospitalized patients, noting that at that time, there was only one specialist in pain and palliative care. Because of the need to improve the multidisciplinary approach to pediatric oncology patients, the Fundación Ayúdame a Vivir pro-Niños con Cáncer de El Salvador (FAV) opened dedicated unit space to provide PPC support to outpatients twice a week; then, early in 2011, support became available on weekdays. 

The HNNBB is the only specialized care center for the pediatric population in El Salvador, providing pediatric oncology care to approximately 200 new cases of pediatric cancer in children younger than 14 years of age each year. The number of cancer patients seen in any given year is approximately 600 patients, including 400 children seen as new referrals or receiving active treatment, and 200 children who have completed follow-up but are in follow-up clinics. In 2014 the in PPC team began seeing children admitted to the general pediatrics, hematology, infectious diseases, nephrology, and other inpatient services. 

Despite space and staffing constraints (only two pain specialists provide services at HNNBB), this service was emulated by other hospitals that are part of the integrated health network in El Salvador. A total of 19 units have been opened throughout the country for provision of pain and symptom management for patients requiring palliative care services. The PPC team at HNNBB has organized four didactic courses in palliative care, which subsequently facilitated the opening of dedicated services throughout the country. [11,12]

## 4. Costa Rica

On 1 October 1990, the Pediatric Palliative Care and Pain Control Clinic began operation at the National Children’s Hospital “Dr. Carlos Sáenz Herrera”—the only pediatric hospital in the country—under the direction of its founder, Dr. Lisbeth Quesada Tristán. 

Dr. Quesada Tristán, a general pediatrician, was awarded a scholarship at the Sloan Kettering Center in New York, New York. She and a nurse, Ada Rogers, established the clinic despite a scarcity of resources. Dr. Quesada’s initial task was to create a multidisciplinary team and educate the health care staff about which children needed palliative care.

At this time, the PPC service was made up of two teams, one located in San José (the capital city) and the other one in Pérez Zeledón (a city in the southern part of the country). The team, which consisted of only one physician and a nurse in 1990, now has 6 physicians, 3 nurses, 4 psychologists, a dietitian, a physical therapist, a respiratory therapist, and a social worker.

The Palliative Care Team members entered into an agreement with the Catholic University of Costa Rica to offer a master’s degree in Palliative Care, which has allowed all the existing team members and many other professionals to acquire a Master’s academic degree. The first fellowship-trained pediatrician graduated from the British Columbia Children’s Hospital and Canuck Place Children’s Hospice in Vancouver, Canada, and was incorporated into the team in 2009, upon graduation from her fellowship in Canada.

The team delivers care in three settings: inpatient consultations, home visits throughout the entire country (remarkably, this has continued uninterrupted despite the COVID-19 pandemic) and a day hospital palliative care unit, where interdisciplinary, intensive outpatient consultation is provided to the child and their family members. These facilities are called Albergue San Gabriel and Albergue Acosta Rúa. 

Approximately 1300 children with life-limiting conditions receive care from the palliative care teams. Of these, 60% have central nervous system conditions, 15% have cancer (including children still receiving disease-directed therapies, and patients in whom active disease-directed therapies have been discontinued, with a focus on the management of pain and other distressing symptoms). Overall, 10% of the patients have cardiopulmonary conditions, and 6% have other neuromuscular diseases. The balance is made up of chromosomal, immunologic, metabolic, and renal conditions.

**Figure 2:**
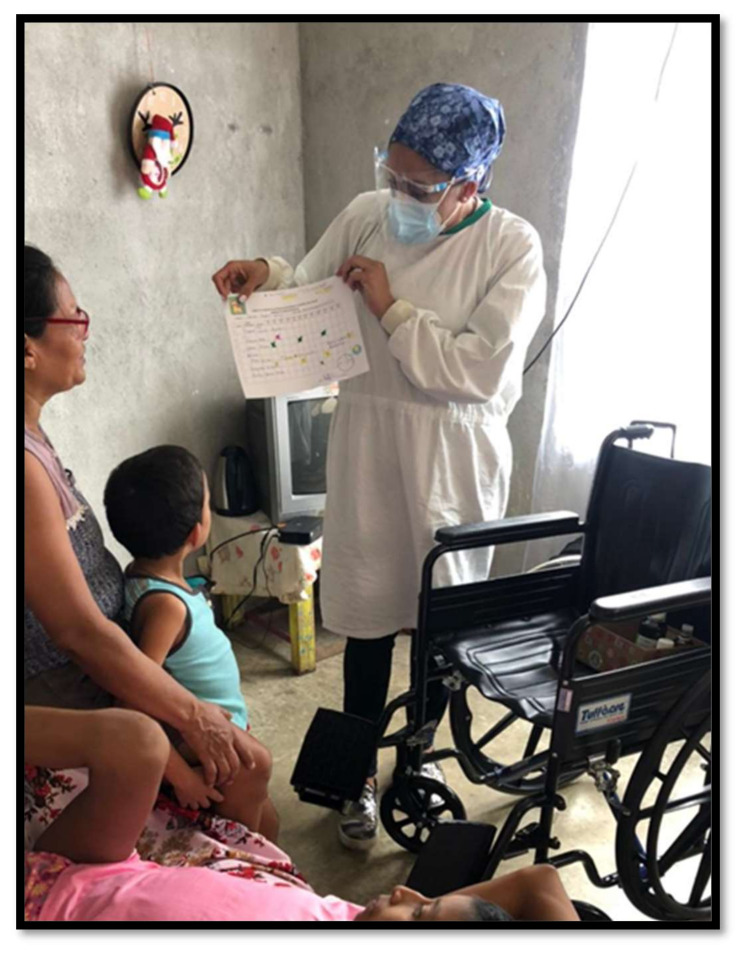
Education to a patient’s family in Costa Rica.

Although Costa Rica also has 23 adult PC clinics accredited by the Ministry of Health, the only PPC program, the one founded by Dr. Lisbeth Quesada Tristan, provides pediatric palliative care to the entire country. In order to achieve its mission of ensuring coverage to every single Costa Rican child requiring palliative care, it is supported by governmental institutions (the Costa Rica Social Security System) and non-governmental institutions (Fundación Unidad Pro-Cuidados Paliativos). Continuous coordination is carried out with all clinical services located in the twenty general hospitals with general and specialty pediatric services, and the nearly 1000 primary care clinics distributed throughout Costa Rica, one of the most comprehensive health care systems in Central America. This network of primary care providers, regional hospitals with pediatric services, and the referral children’s hospital in San José facilitates continuity of care, even in remote or difficult-to-access areas where the country’s indigenous populations live (Figure 2).

The Fundación Unidad Pro-Cuidados Paliativos, established in 1992, manages all the administrative aspects of this non-governmental agency, including the hiring of clinical and support staff, provision of resources that are necessary for home visits including supplies required by the children, and financial aid for the most impoverished families. Continuous medical education opportunities have been created for health care professionals, including pediatricians by the Fundación. The PPC services have expanded to include acute and chronic pain clinics that are also attended by children who don’t have immediately life-threatening conditions, the Integrative Medicine Clinic, the Perinatal Palliative Care Clinic, and the Grief and Support Program for parents and relatives. 

The current challenges of the existing team are that, for example, the National Children’s Hospital has 19 pediatric subspecialties. There is concern that some of the children attending these subspecialty services could potentially benefit from PPC support utilizing a concurrent care model. Efforts are therefore underway to train more pediatricians in PPC, nationally and internationally.

## 5. Honduras

Honduras is a country located in Central America with a population of about 10.1 million people. It has an estimated poverty rate of 70%—including 53.4% of Honduran inhabitants living in extreme poverty in 2021 [13].

It has been estimated that a new pediatric cancer diagnosis is made on any given day. Unfortunately, the unstable socioeconomic conditions of the country make it difficult for pediatric cancer patients to receive optimal disease-directed treatment and palliative support. Sadly, the lack of financial resources has forced the public health system to prioritize disease-directed interventions to children judged to have a better overall prognosis. The unfortunate result of this strategy is a large cohort of seriously ill children with unmet palliative care needs. The Honduran Foundation for Children with Cancer was therefore created in 2017 with the goal of training pediatric oncologists in basic palliative care, working in close collaboration with the Hospital Escuela, the primary teaching hospital in Honduras.

Expansion of this program through ambulatory visits has facilitated inpatient and outpatient services using an interdisciplinary team that consists of a palliative care doctor, a palliative care nurse, a psychologist, and a social worker.

The pediatric hematology-oncology unit at the Hospital Escuela has 24 beds for children requiring inpatient care. 3 additional beds are reserved for children with difficult-to-control symptoms that require advanced levels of palliative care. One room is reserved for children who are actively dying. The palliative care outreach program based at the Hospital Escuela was strengthened by assigning a team member to provide telemedicine, primarily through weekly phone calls. That way, the team can remain abreast of the overall condition of each patient by utilizing the Lansky Scale, and also to help identify physical, psychological, spiritual, and economic needs in a timely fashion. Unfortunately, staffing constraints limit home visits to areas that are close enough that the team can make the round trip in a day. 

Of note, the foundation is building a new medical center for children with cancer in Tegucigalpa. This new facility, remarkably, has been designed with ten inpatient beds specifically designated for palliative care.

Although there is clearly a lot of work to be done, we are proud of our many achievements:

1. The formation of the palliative care team,
2. Daily clinical rounds by a palliative care physician and a psychologist,
3. Home visits, which have not stopped despite the COVID-19 pandemic.
4. Overall, 100% of cancer patients who have died have had some intervention by our PPC team,
5. Educational talks on PC continue in the main hospitals throughout the country,
6. Psychological follow-up from the beginning diagnosis, regardless of curative intent,
7. Accompaniment the patient and the family members throughout the continuum of care, including the bereavement process,
8. Administration of pain medications, when indicated, through the use of an elastomeric pump,
9. Each family is given medicine and sanitizing kits, food, access to recreational activities that are carried out near their home, sponsorship for birthday celebrations, wish fulfillment, and music therapy,
10. The social worker conducts an interview with the family looking for opportunities to improve their home conditions.

**Figure 3:**
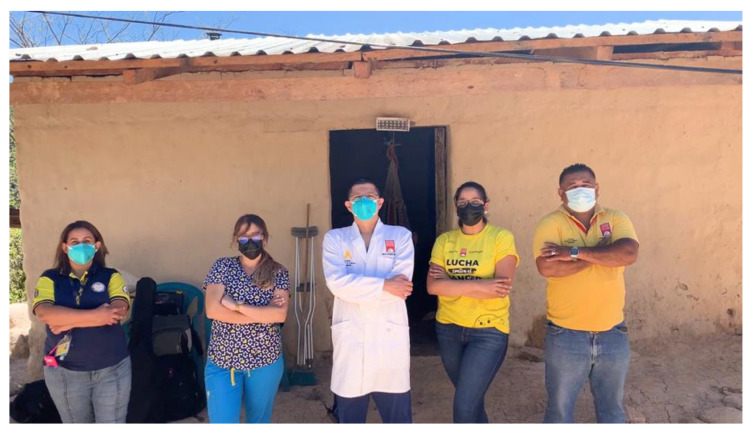
PPC Team on a home visit in Honduras.

This program has visited families in their homes in Tegucigalpa and throughout the rest of the country; capacity is growing despite the pandemic, primarily in the inpatient setting [14]. See Figure 3.

## 6. Panamá

Legislation regarding guaranteed access to palliative care has been in place since 2003 [15] as part of the National Palliative Care Program. Prior to the implementation of this legislation, palliative care was primarily provided through the “Grupo de Amigos del Niño con Enfermedad Terminal (Group of Friends of The Terminally Ill Child)“, which was founded in 1998 by a mental health nurse, Jeanette Precilla. 

Subsequently, a hospital committee on pediatric palliative care was created by pediatric bioethicist Claude Verges de López. It is estimated that, on a national level, 4700 patients are receiving palliative care. Of these, approximately 4.5% are under the age of 18. 

Approximately 211 children and their families are referred to our specialty-level pediatric palliative care each year. Babies who are referred usually suffer from congenital malformations, hereditary diseases, or disorders of the nervous system. Older children are more likely to have sequelae of severe accidents, or malignancies. 

The National Palliative Care Program has put together teams consisting of a doctor, a nurse, and a social worker for care of patients with high symptom burdens, and during end-of-life. The three main pediatric hospitals in Panama now have pediatric palliative care teams with expertise in pain management and the management of other distressing symptoms that occur at the end of life. 

These teams also help mediate difficult conversations when they are needed, and to provide psychological and spiritual support as required by patients and their families. In the case of *Hospital del Niño*, the national referral children’s hospital, the team is composed of two nurses, a pharmacist, a social worker, a spiritual support provider, a hematologist, a dedicated pediatrician, and a pediatric palliative care specialist. 

Law 16, passed in 2016, updates and guarantees the availability of controlled pain medicines—quite problematic throughout Latin America—for use in patients needing them in Panama [2]. In 2018, the National Palliative Care Program published a Guía de Manejo (Management Guidelines) to facilitate the management of the most common problems that occur in the patients receiving palliative care [16].

The team has been collaborating closely with other institutions to facilitate palliative primary care for clinicians who are responsible for the outpatient and home consultations in the various health regions. The challenge imposed by the COVID-19 pandemic inspired our efforts to improve the quality of PC using novel strategies. A picture book with instructions on the care of ill family members is in process, in addition to educational videos. A pilot plan to help empower the child’s caregiver has been launched. In this program, vital information is shared in real time through instant messaging [17].

The challenge that we anticipate in the near future is a transition medicine clinic for adolescents, with a focus on their quality of life, when advanced oncological disease has been diagnosed. We are hoping to achieve this by working closely with other pediatric subspecialties, advocating for more patient-focused and family-centered care in hospital settings, and helping direct community efforts that can help these children and their families achieve a better quality of life, thus making our palliative care team even more effective.

## 7. Dominican Republic

The first PPC in the country, called the Palliative Care & Metronomic Therapy Program in the oncology unit of Dr. Robert Reid Cabral Children’s Hospital, was established in January 2012. This is the largest pediatric level 3 hospital of the Dominican Republic. This program was founded and is currently directed by Dr. Wendy Gómez García [18], in response to the lack of protocols in place to offer care to palliative patients (children or adults). There is also a lack of educational programs, and there are no formal palliative care training programs [19].

The program has provided specialized palliative care (after all disease-directed interventions have been exhausted) principally in the setting of metastatic or refractory disease after the failure of 1 or 2 front-line protocols. Extreme poverty contributes to delays in diagnosis and complicates treatment adherence. Disease recurrences or progression occur as locally available therapeutic options are exhausted. Many of the patients referred to our program, therefore, have advanced disease and usually have a high symptom burden. Once the patients are referred to the specialized PPC program, a series of outpatient follow up consultations is begun, so that patients and their families are able to receive advice, practical and bereavement support, in addition to the management of pain and other distressing symptoms. 

**Figure 4:**
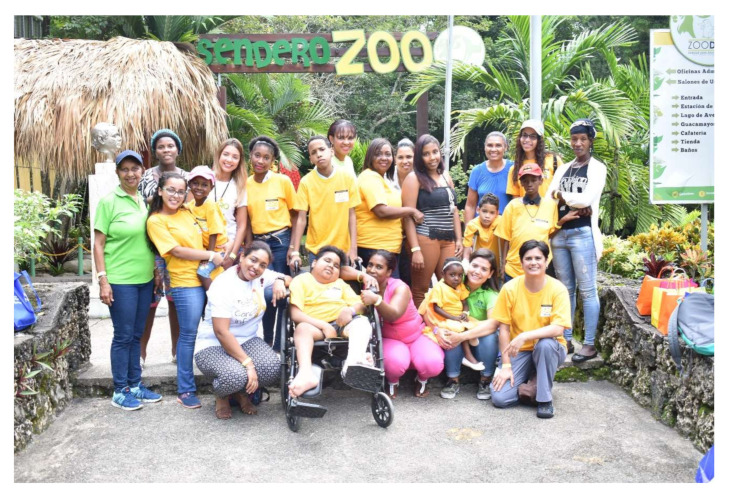
Visit to the zoo with patients, families, team and volunteers of Dominican Republic’s Palliative Care Program.

The PPC team offers longitudinal care, 24/7 emergency phone line support, metronomic chemotherapy for disease control, when indicated, and some financial support through FACCI, Fundación Amigos Contra el Cáncer Infantil (Friends Against Childhood Cancer Foundation), which has also created pain and symptom control protocols and guidelines, individualized care plans, and facilitated access to local resources. A dedicated team of volunteers is an invaluable asset to our team and help the clinical team to coordinate visits and playful walks (See Figure 4) [20].

**Figure 5:**
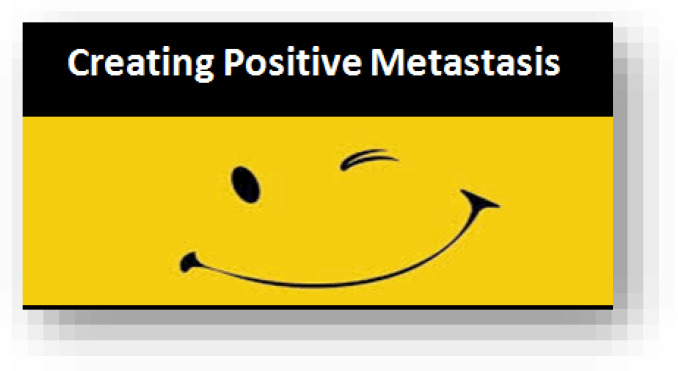
Creating Positive Metastasis Logo.

PPC team has organized a series of national and international conferences, offered to provide continuing medical education for medical students, primary care pediatricians, and hematology/oncology specialists, with the vision of “Creating Positive Metastasis”, (see Figure 5) which seeks to find meaning in the catastrophic reality that pediatric cancer presents, modeling a positive attitude when sharing information and knowledge about palliative care, promoting resilience, and helping to contribute to the global dimensions of these diseases [21,22,23,24,25].

On 11 June 2019, the First International Seminar on Pediatric Palliative Care and Pain Management of the Dominican Republic was held in the capital city of Santo Domingo. This academic stage brought together leaders in the field, with the objective of sharing knowledge of the key concepts of PPC, pain management, management of other distressing symptoms, and strategies to improve the quality of life of patients facing catastrophic diseases, including cancer [26,27].

The PPC have been quite gratified to see incredible advances, which translate into improved quality of life of children with cancer; empowerment from FACCI Foundation has led to improvement in the structure and staffing of our programs. We are glad that the suffering of children and their families has been reduced. One thing is for sure: the program will continue creating positive metastasis [28,29,30].

## 8. Haiti

The Oncology Unit at Saint Damien Hospital (SDH) was founded in 2004. Before then, the pain treatment in the context of palliative care was offered only on a case-by-case basis. In 2014, the head nurse of the oncology unit received PPC training in Guatemala. Two years later, in 2016, a pediatrician completed PPC training. This made it possible for the PPC team to finally consolidate with the formation of an interdisciplinary team for provision of support to children and families.

At the beginning, the supply of medications for pain management was a challenge in Haiti. Since 2017, however, thanks to the ongoing efforts of the interdisciplinary team, and the support of the Spanish NGO “Palliativistas Sin Fronteras” (Palliative Care without borders), the program has had access to some analgesics, including oral morphine and fentanyl patches. Unfortunately, however, we have been unable to completely meet pain medication demand.

PPC is essential in Haiti, due to the fact that a very high proportion of children with cancer present for care at an advanced stage of their illness, when there is little, if any, chance of recovery. The program has enrolled more than 390 new children with cancer in the program, noting that 123 (over 31% of newly diagnosed pediatric malignancies) of these children have been managed exclusively with symptom control, because of the advanced stage of disease upon presentation.

Pediatric palliative care, integrated from the time of diagnosis of the disease, provides significant benefits to the child and his family, from all standpoints. Nevertheless, patient attrition occurs for a variety of factors, including low educational level, religious and cultural beliefs that impede access to traditional medical care, extreme poverty, and other complex social factors. Sadly, 24 children abandoned their palliative care follow up, possibly due to a combination of escalating political local instability and the effects of the COVID-19 pandemic.

In Haiti, as soon as an oncological diagnosis is made, the team meets with the whole family to discuss treatment approaches, adapting language to the cultural and academic levels of the parents. Unfortunately, denial, the inability to accept the diagnosis, places the child on the track of abandonment. People’s religious and cultural beliefs about cancer have long been identified as a barrier. Some families prefer to let their children die in a religious or voodoo priest’s home instead of accepting that the child is seriously ill and needs medical care. Other factors that contribute to abandonment or refusal of treatment are socioeconomic and demographic problems, even though parents do not have to pay for the treatment. In some cases, parents are not comfortable receiving care in the city, because many of them come from rural areas and would prefer to have additional comforts that PPC cannot currently provide [31,32].

## 9. Measures to Mitigate the Abandonment of Cancer Treatment and Existing Measures in Latin America 

Treatment abandonment or attrition in underdeveloped countries determines the strong difference observed in survival curves, when compared to more prosperous areas of the world. It is responsible for the preventable loss of many lives. Costa Rica has a very clear legal framework in which the “Childhood and Adolescence Code” allows inter-institutional coordination to prevent treatment abandonment. This can serve as a model for other countries in the Americas. Psychosocial evaluation is carried out upon diagnosis, to detect risk factors for abandonment. If a child misses an appointment, failure to attend the clinic or hospital is reported to the social work team, and an alert is activated so that contact with the parents is established to determine the cause of the missed appointment. In addition, special housing (i.e., the Albergues) has been arranged near the hospital, so that one of the parents and the patient can stay on site as long as necessary, until the treatment is finished. The neediest families are helped both by said association and by the Costa Rican Social Security Fund, which covers travel expenses and even basic needs. All these efforts have led to, with the sole exception of 2005, not a single family abandoning their cancer care.

In Guatemala, close collaboration between oncologists and the Palliative Care team has generated unexpectedly positive results, especially in children with overall good prognosis, through the prevention of attrition from disease-directed therapies, since it is known that poor adherence or abandonment cause treatment failures. The program seeks to improve communication, facilitate social programs, and integrate psychology care, in order to address the causes that lead to treatment abandonment. 

The team of the National Pediatric Oncology Unit, through the creation of different programs that originated from the Medicina Integral (Integrative Medicine) team, reduced the abandonment rate from before 2005 of 20%, to less than 1%, through the concerted efforts of everyone involved. The programs have been much more successful than initially expected, through the implementation of the basic tools of Palliative Care, including impeccable communication, optimal use of existing resources, and teamwork. 

A few of our successful initiatives include:

1. Financial support to low-income families identified through assessment of each family to ensure proper allocation of funds, transportation to families from rural areas, Self-care strategies for family members and other caregivers, supplemental food for the families staying at home, and other initiatives.
2. Work in conjunction with community leaders, and state organizations for the prompt referral of cases that need legal intervention to ensure treatment adherence.
3. Emotional support of families for compliance with indicated treatments, regardless of prognosis.
4. Provision of accommodation for families who come from far distances for hospital care.

## 10. Conclusions

Palliative care is a specialty dedicated to improve the quality of life for patients with life-threatening conditions; in this case, particularly in the pediatric oncology population. Although the access to pediatric palliative care services is limited by the number of programs, the care of the patients that require this multidisciplinary approach continues to increase. We presented multiple successful stories that ranged from programs such as the National Pediatric Oncology Unit in Guatemala (UNOP), which also provides inpatient hospice care, to other context-appropriate palliative care teams in El Salvador, Costa Rica, Panama, Dominican Republic, and Haiti. 

We are very pleased that every single country which came together for the foundation of AHOPCA (the Association of Central American Pediatric Hematologists and Oncologists) has found a way to start their very own programs to very successfully offer local support. It is certain that in Central America, although palliative care practice has just begun in some countries, many patients, families, and teams have benefited from the improved quality of life that is only possible through an integrated, holistic approach to care.
